# Factors related to steroid treatment responsiveness in thyroid eye disease patients and application of SHAP for feature analysis with XGBoost

**DOI:** 10.3389/fendo.2023.1079628

**Published:** 2023-01-31

**Authors:** Jungyul Park, Jaehyun Kim, Dongman Ryu, Hee-young Choi

**Affiliations:** ^1^ Department of Ophthalmology, Pusan National University Hospital, Busan, Republic of Korea; ^2^ Biomedical Research Institute, Pusan National University Hospital, Busan, Republic of Korea; ^3^ Medical Research Institute, Pusan National University, Busan, Republic of Korea; ^4^ Department of Ophthalmology, School of Medicine, Pusan National University, Busan, Republic of Korea

**Keywords:** extreme gradient boost, XGBoost, machine learning, graves orbitopathy, thyroid eye disease, novel risk factors

## Abstract

**Introduction:**

The primary treatment for active thyroid eye disease (TED) is immunosuppressive therapy with intravenous steroids. In this study, we attempted to predict responsiveness to steroid treatment in TED patients using eXtreme Gradient Boosting (XGBoost). Factors associated with steroid responsiveness were also statistically evaluated.

**Methods:**

Clinical characteristics and laboratory results of 89 patients with TED who received steroid treatment were retrospectively reviewed. XGBoost was used to explore responsiveness to steroid treatment, and the diagnostic performance was evaluated. Factors contributing to the model output were investigated using the SHapley Additive exPlanation (SHAP), and the treatment response was investigated statistically using SPSS software.

**Results:**

The eXtra Gradient Boost model showed high performance, with an excellent accuracy of 0.861. Thyroid-stimulating hormone, thyroid-stimulating immunoglobulin (TSI), and low-density lipoprotein (LDL) cholesterol had the highest impact on the model. Multivariate logistic regression analysis showed that less extraocular muscle limitation and high TSI levels were associated with a high risk of poor intravenous methylprednisolone treatment response. As a result of analysis through SHAP, TSH, TSI, and LDL had the highest impact on the XGBoost model

**Conclusion:**

TSI, extraocular muscle limitation, and LDL cholesterol levels may be useful in predicting steroid treatment response in patients with TED. In terms of machine learning, XGBoost showed relatively robust and reliable results for small datasets. The machine-learning model can assist in decision-making for further treatment of patients with TED.

## 1 Introduction

Thyroid eye disease (TED) is a condition in which an immunological response to soft tissue in orbit creates severe patient discomfort, necessitating anti-inflammatory treatment if necessary. The current understanding of the underlying mechanism suggests that the interactions between the autoimmune response and thyroid-stimulating hormone (TSH) receptor-expressing orbital fibroblasts in orbit are the primary pathogenesis of TED. Recent research indicates that insulin-like growth factor-1 receptors play a synergistic role in TSH receptor (TSHR)-initiated signaling, in addition to TSHR activation (1, 2).

Immunosuppressive treatment with intravenous steroids has been the major treatment for active TED, and pulsed intravenous methylprednisolone has been shown to be effective. Radiation and immune modulators, such as rituximab and teprotumumab, can be employed to manage TED (3). However, as recommended by a recent consensus statement issued by the European Group on Graves Orbitopathy (EUGOGO), intravenous methylprednisolone (IVMP) remains the first-line treatment for moderate-to-severe and active TED (4).

According to previous research, up to 20%–25% of clinically active, moderate-to-severe TED patients may not respond to steroids and/or relapse after treatment discontinuation (5). The clinical activity score (CAS), TSHR antibody (TRAb) level, triglyceride level, and disease duration are reportedly associated with responsiveness to IVMP treatment (6, 7). However, the effect of these factors on IVMP remains controversial. Furthermore, systemic steroid medication may be advantageous in patients with TED; however, it should be used with caution because of possible systemic side effects (4). Moreover, the mechanism of action of high-dose steroids is unknown and must be weighed against its harmful effects (8). Therefore, predicting the steroid response in patients should be strongly considered.

In this study, we used eXtreme Gradient Boosting (XGBoost), a scalable machine-learning system for tree boosting, to predict steroid treatment responses in patients with TED. The XGBoost system is an open-source package. The usefulness and superiority of the system have been widely recognized in several machine-learning challenges (9). It uses a gradient-boosting framework to create machine learning with excellent efficiency, flexibility, and probability (9). To improve our model, we applied the synthetic minority oversampling technique (SMOTE) and feature selection (FS) to detect essential features for classification. Finally, the FS-based XGBoost model proposed the feature importance associated with responsiveness in detail with SHapley Additive exPlanations (SHAP).

To the best of our knowledge, this is the first time that the gradient boosting machine, the XGBoost system, has been used in this field. According to this study, despite the small amount of data utilized for training, the FS-based XGBoost model showed stable and high performance. Factors related to steroid responsiveness were investigated using univariate and multivariate statistical methods.

## 2 Methods

### 2.1 Patient recruitment

This retrospective study was conducted in accordance with the principles of the Declaration of Helsinki. This study was approved by the Institutional Review Board (IRB) of Pusan National University Hospital (IRB No. 2112-006-109), South Korea. Owing to the retrospective nature of the study, the IRB waived the need for patient consent.

In this study, patients with TED were enrolled at the Oculoplasty Clinic of Pusan National University Hospital between March 1, 2016 and December 31, 2020. When both eyes of a participant were eligible, the more severe eye was chosen for inclusion and data analysis.

Among 287 patients diagnosed with TED, 89 were enrolled and divided into two groups. One group showed responsiveness to steroid treatment (responsive) and the other showed no response to steroid treatment (unresponsive). Patients who were enrolled in this study satisfied all the following conditions: (1) a TED diagnosis based on the EUGOGO consensus and (2) patients who were in the active phase and moderate to severe category based on the CAS. The exclusion criteria were as follows: (1) treatment with other immunosuppressive therapies, decompression surgery, or radiotherapy within the previous 3 months or during IVMP therapy; (2) not having completed the full course of the IVMP treatment regimen; (3) incomplete ophthalmic assessment data and/or essential laboratory tests including free T4, T3, TSH, TRAb, and thyroid-stimulating immunoglobulin (TSI); and (4) patients with a previous medical history of glaucoma, diabetic retinopathy, maculopathy, or strabismus.

The treatment protocol was as follows: IVMP was administered by an endocrinologist for 12 weeks. MP was injected weekly on the same day at a 0.5-g dose and a 0.25-g dose for the remaining 6 weeks. We monitored liver function and blood glucose levels regularly. Anti-thyroid drugs or thyroxine were used to restore and maintain euthyroidism. Individuals who smoked were advised to abstain from smoking.

### 2.2 Data collection and outcome evaluation

One ophthalmologist in the oculoplastic division described all ophthalmic examinations in the medical records. Two investigators in the oculoplastic division analyzed the electronic medical records of these patients to determine their eligibility for the study. Demographic and biochemical data and additional pertinent clinical data were evaluated. TRAb levels were measured using the third-generation thyrotropin-binding inhibitor immunoglobulin assay, which inhibits the binding of labeled thyroid-stimulating autoantibody (TSAb) (monoclonal Ab clone #M22) to the TSH receptor. The TSI was measured using a thyroid-stimulating immunoglobulin (TSI) bioassay, which measures cyclic adenosine monophosphate production after TSAb binds to the TSH receptor. Smoking status was categorized as never smoked, ex-smoker, or current smoker. In the statistical analysis, we classified ex-smokers and never-smokers as nonsmokers.

**Figure 1:**
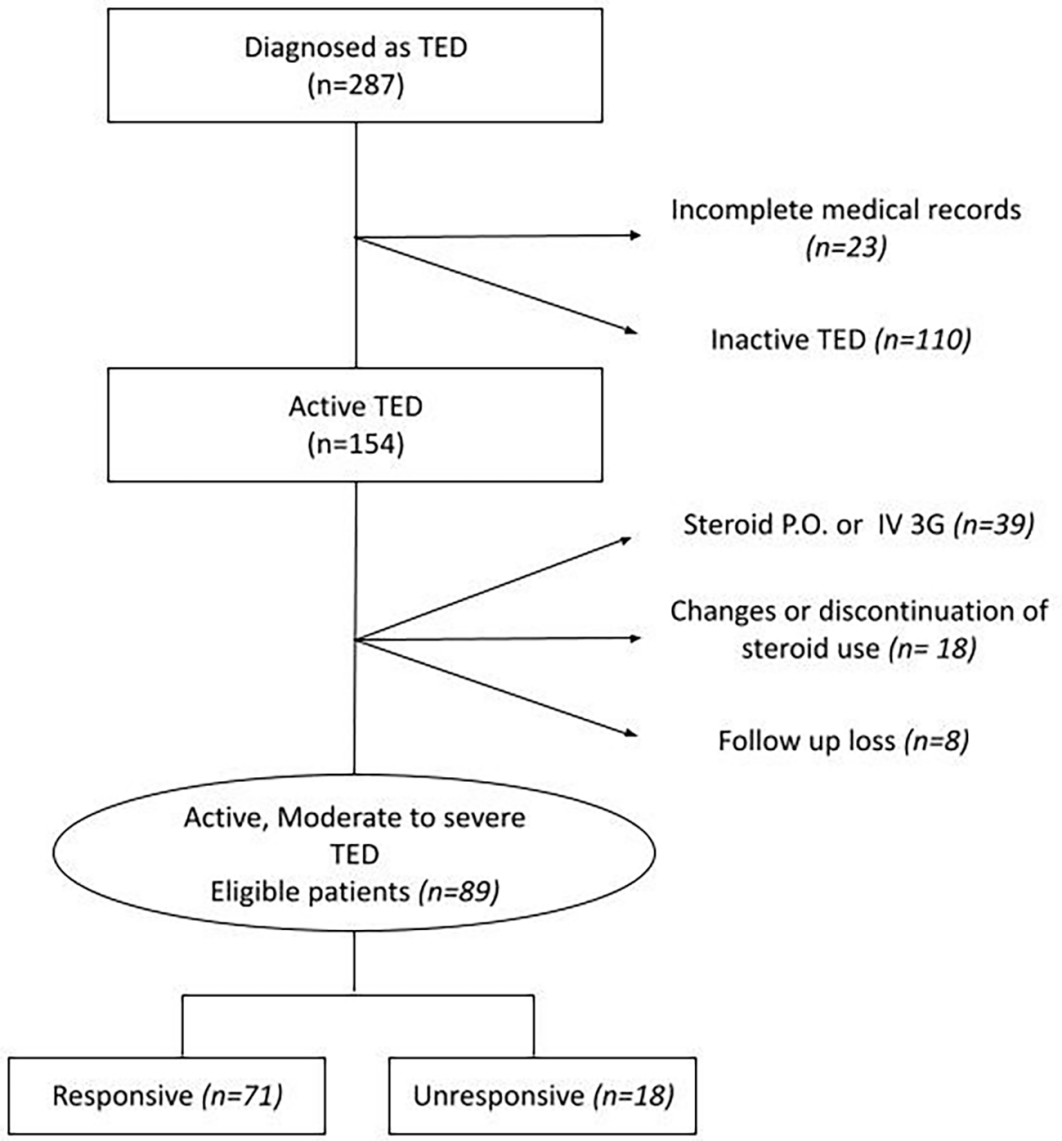
Flow chart of the data extraction. P.O., per oral; TED, thyroid eye disease; IV, intravenous; 3G, 3 grams.

The presence of at least two of the following five ophthalmic parameters was defined as the response after full doses of IVMP treatment: 1) a reduction of at least 2 mm in proptosis; 2) a reduction of CAS by at least 2 points or <3/7; 3) an increase in visual acuity of at least one Snellen line; 4) improvement in diplopia (decrease in Gorman degree); and 5) no recurrence or additional radiation therapy at least 6 months after IVMP treatment. Based on these assessments, we divided the patients into “responsive” and “unresponsive groups.” Patients who received additional radiation therapy after IVMP treatment or decompression surgery during IVMP treatment were classified as “unresponsive.” Detailed patient recruitment flow charts are illustrated in **Figure 1**.

### 2.3 Data preparation: SMOTE and FS

We applied the synthetic minority oversampling technique and FS method to overcome the shortage of datasets and minimize computational complexity. We constructed a supervised machine-learning classification model using XGBoost in the following manner: 1) columns in the dataset were selected using the statistical hypothesis test (categorical variable: chi-squared or the Fisher exact test; continuous data: t-test or the Mann–Whitney U test). This eliminates redundant and irrelevant features to simplify the training model. 2) Next, FS was performed based on feature importance using a combination of random forest and XGBoost. Random forest and XGBoost FS methods have been applied in various studies and have shown good performance (10–12). We collected 17 top-ranked features in descending order of importance to describe responsiveness. Data preparation is performed by reducing data dimensionality and creating a model with only important features. 3) Finally, we applied SMOTE to increase the number of under-presented cases, as reported in another study (13). SMOTE takes each data point from a minority class and generates new members along a line that connects them to their k-nearest neighbors (13, 14). We did not employ data normalization and one-hot encoding in our model to prevent performance degradation (9).

**Figure 2:**
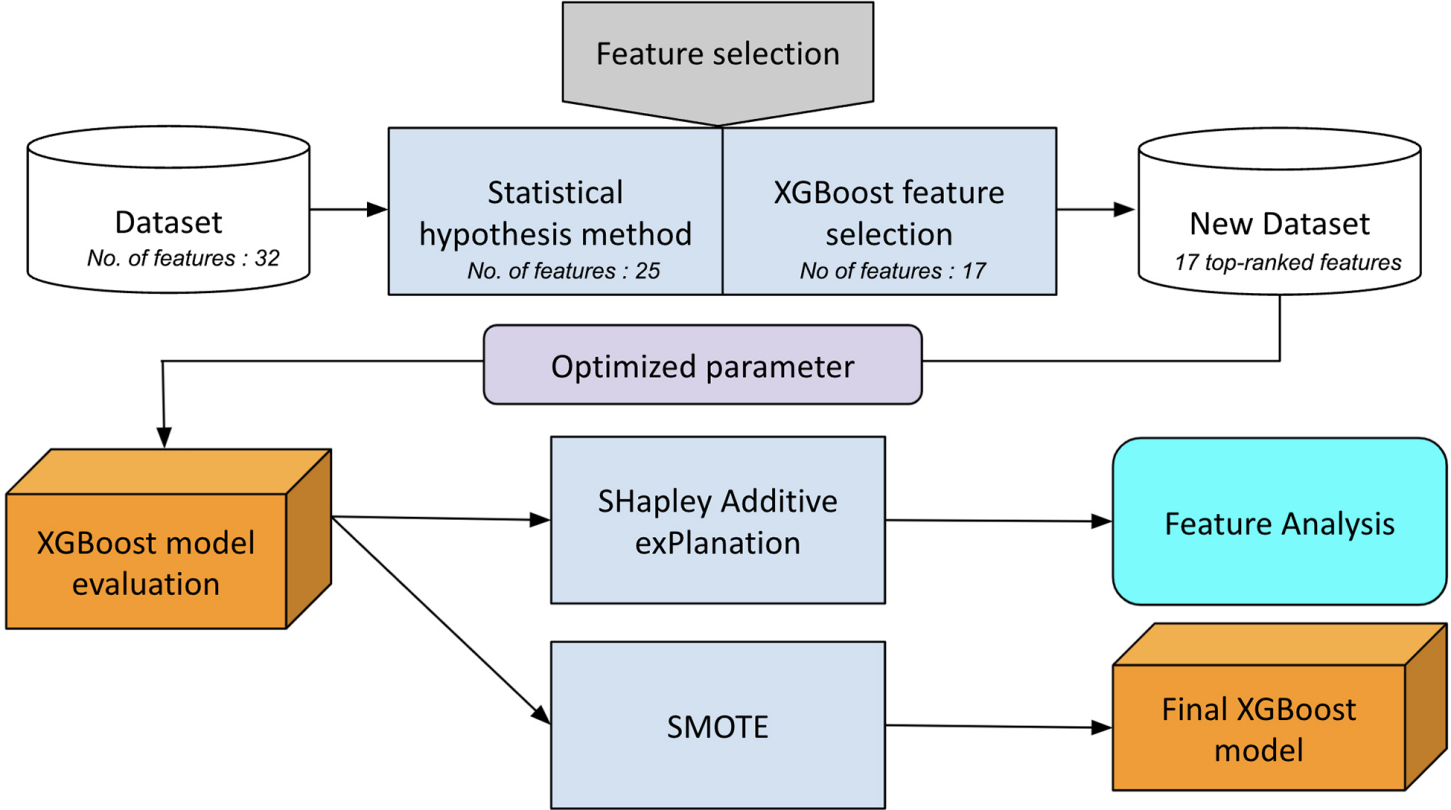
Flow chart of the hybrid feature selection to construct the final XGBoost predictive model.

In our experiments, the scikit-learn machine-learning framework was used to implement the XGBoost. During the 10-fold cross-validation, we optimized the parameters of the model. The hyperparameter values selected in our model are as follows: booster: gbtree; n_estimators: 300; max depth: 5; loss function binary: logistic; subsample = 1; lambda = 0.7; alpha = 0.15; and learning rate = 0.03. A flowchart of the experimental design is shown in **Figure 2**.

### 2.4 XGBoost classifier

We underwent training for the state-of-the-art scalable boosting machine-learning method called XGBoost (9). Python language version 3.8 was used, and the hardware environment was an AMD Ryzen 7 8-core processor with 32 GB RAM and GeForce 2070Ti video cards (NVIDIA, Santa Clara, CA, USA). XGBoost is a tree-boosting technique that is highly scalable and accurate. Traditional gradient boosting incorporates the following components: (1) a regularization term into the objective function, (2) an approximate split using a weighted quantile sketch, (3) a sparsity-aware split function for parallel tree learning, and (4) out-of-core computation and cache-aware learning.

All participants were randomly assigned to either the training set (80%, 71 of 89 patients) or the test set (20%, 18 of 89 patients). The training dataset was not large, but XGBoost had significant adaptations to reduce overfitting and was applied to a wide range of issues. Other regularization approaches, such as shrinkage and column subsampling, were also included. Shrinkage reduces the impact of individual trees while allowing subsequent trees to improve the model. Column subsampling was initially widely utilized in random forests, although it has never been employed for tree boosting (15). According to feedback, column sub-sampling was applied, preventing overfitting compared to row sub-sampling. Furthermore, it also accelerates the parallel technique detailed in the subsequent sections of this paper.

### 2.5 Model evaluation and interpretation

The CV mean accuracy was used to evaluate the prediction performance of our model. To interpret the results and importance of each feature, we applied SHAP. This allowed us to provide a powerful and insightful measure of the relevance of a feature in a model. XGBoost model was compared with other machine learning algorithms and linear regression model which is traditional statistical model. We compared the XGboost model before and after the SMOTE statistically with CV mean accuracies.

### 2.6 Statistical analysis

The Kolmogorov–Smirnov test was used to determine the normality of the data distribution. Continuous data were compared using Student’s t-test or Mann–Whitney U test. Categorical variables were compared using the chi-square test or Fisher’s exact test. Univariate logistic regression analysis was performed to determine the significance of each variable. Significant variables identified in the univariate analysis were then subjected to multivariate logistic regression analysis to identify the independent factors associated with responsiveness to IVMP treatment. Statistical Package for the Social Sciences (SPSS) software (version 22.0; SPSS, Chicago, IL, USA) was used for all statistical analyses. Statistical significance was set at P<0.05.

## 3 Results

### 3.1 Patients characteristics

All variables, including the demographic, clinical, and biochemical characteristics, are presented in **Table 1**. Among the 89 active, moderate-to-severe TED patients who were administered 4.5 g of methylprednisolone intravenously, 71 (79.8%) were included in the responsive group, and 18 (20.1%) were included in the responsive and unresponsive groups. When comparing never smokers to ex-smokers in the responsive group, the response rate was significantly higher in former smokers than in never smokers (*P* = 0.029, 100% vs. 71.2%). When we classified ex-smokers as never smokers, there was no difference in responsiveness between never-smokers and current smokers (*P* = 0.377) (data not shown). CAS was higher in the unresponsive group, but the difference was not statistically significant (*P* = 0.065), and extraocular movement limitation was more severe in the responsive group (*P* = 0.054). Overall, there were no significant differences between the two groups, except for the smoking status.

**Table 1:** Demographic characteristics of the responsive and unresponsive group.

	Total	Response to steroid treatment		
		Responsive	Unresponsive	p-value
**Number of patients (%)**	89	71 (79.8)	18 (20.1)	
**Age (years, mean ± SD)**	51.34 ± 13.79	51.25 ± 13.67	51.67 ± 14.62	0.910^a^
**Sex (male:female)**	39:50	32:39	7:11	0.637^b^
**Height (cm, mean ± SD)**	163.35 ± 7.37	163.38 ± 7.26	163.22 ± 8.04	0.933^a^
**Weight (kg, mean ± SD)**	62.67 ± 10.18	62.45 ± 10.79	63.55 ± 7.46	0.683^a^
**BMI (kg/m^2^, mean ± SD)**	23.39 ± 2.69	23.28 ± 2.89	23.81 ± 1.66	0.320^a^
**Thyroid eye disease type (fat dominant: muscle dominant)**	27:62	22:49	5:13	0.791^b^
**Thyroid eye disease symmetry (both: asymmetric: unilateral)**	57:28:4	44:23:4	13:5:0	0.729^d^
**Graves’ disease duration (months, mean ± SD)**	32.01 ± 59.47	33.70 ± 59.87	25.33 ± 59.07	0.289^c^
**Thyroid eye disease duration (months, mean ± SD)**	17.00 ± 35.77	19.52 ± 39.61	7.06 ± 5.68	0.566^c^
**Family history (present: absent)**	20:69	15:56	5:13	0.540^d^
**Smoking (never smoker: ex-smoker: current smoker)**	52:13:24	38:13:20	14:0:4	
**never smoker: current smoker**		37:21	15:3	0.119^b^
**never smoker: ex-smoker**		37:13	15:0	*0.029^b^
**ex-smoker: current smoker**		13:21	0:3	0.538^b^
**CAS (mean ± SD)**	3.98 ± 1.02	3.87 ± 0.97	4.39 ± 1.14	0.065^c^
**Gorman (mean ± SD)**	1.22 ± 1.32	1.35 ± 1.32	0.72 ± 1.23	0.054^c^
**Visual acuity (OD) (mean ± SD)**	0.12 ± 0.20	0.12 ± 0.20	0.14 ± 0.23	0.672^c^
**Visual acuity (OS) (mean ± SD)**	0.12 ± 0.26	0.12 ± 0.28	0.12 ± 0.21	0.959^c^
**Intraocular pressure (OD) (mmHg, mean ± SD)**	16.16 ± 3.09	16.24 ± 3.20	15.83 ± 2.66	0.613^a^
**Intraocular pressure (OS) (mmHg, mean ± SD)**	16.23 ± 3.17	16.28 ± 3.20	16.02 ± 3.14	0.761^a^
**Exophthalmos measurement (OD) (mm, mean ± SD)**	18.12 ± 3.03	17.90 ± 3.13	18.97 ± 2.44	0.165^c^
**Exophthalmos measurement (OS)(mm, mean ± SD)**	18.19 ± 2.76	17.94 ± 2.80	19.19 ± 2.43	0.098^c^
**Difference in proptosis (mm, mean ± SD)**	1.28 ± 1.12	1.35 ± 1.15	1.00 ± 0.97	0.229^c^
**Extraocular movement limitation (mean ± SD)**	1.44 ± 1.44	1.60 ± 1.50	0.81 ± 0.99	0.054^c^
**Visual field index (OD) (%, mean ± SD)**	94.40 ± 11.91	95.10 ± 7.65	91.67 ± 21.98	0.450^c^
**Visual field index (OS) (%, mean ± SD)**	94.25 ± 13.97	94.67 ± 11.69	92.58 ± 21.07	0.484^c^
**Free T4 (ng/dL, mean ± SD)**	1.39 ± 0.61	1.41 ± 0.64	1.33 ± 0.45	0.818^c^
**TSH (mIU/L, mean ± SD)**	3.25 ± 10.03	2.50 ± 5.03	6.21 ± 20.13	0.072^c^
**T3 (ng/dL, mean ± SD)**	147.82 ± 87.07	148.71 ± 93.01	144.32 ± 60.08	0.806^c^
**TSH receptor antibody (IU/mL, mean ± SD)**	13.49 ± 13.40	12.72 ± 13.00	16.55 ± 14.88	0.299^c^
**Thyroid-stimulating immunoglobulin (%, mean ± SD)**	397.45 ± 166.31	383.15 ± 174.34	453.87 ± 117.46	0.107^a^
**Total cholesterol (mg/dL, mean ± SD)**	186.71 ± 35.38	186.85 ± 37.05	186.12 ± 28.70	0.938^a^
**LDL cholesterol (mg/dL, mean ± SD)**	114.84 ± 30.91	115.96 ± 32.83	110.43 ± 21.93	0.501^a^
**HDL cholesterol (mg/dL, mean ± SD)**	59.41 ± 19.14	58.40 ± 15.67	63.39 ± 29.38	0.980^c^
**Triglyceride (mg/dL, mean ± SD)**	165.31 ± 116.07	174.05 ± 125.52	130.83 ± 57.27	0.146^c^

BMI, body mass index; CAS, clinical activity score; TSH, thyroid-stimulating hormone; LDL, low-density lipoprotein; HDL, high-density lipoprotein; OD, oculus dexter; OS, oculus sinister; SD, standard deviation.

Values are presented as mean ± standard deviation

^*^Statistically significant values with p <0.05.

a Student’s t-test,

b chi-squared test,

c Mann–Whitney U test,

d Fisher’s exact test.

### 3.2 Results of the univariate and multivariate analysis

The summary statistics of the univariate and multivariate analyses between the responsive and unresponsive groups are shown in **Table 2**. As shown in **Table 2**, univariate logistic regression analysis showed that only extraocular movement limitation was significantly associated with responsiveness after IVMP treatment (odds ratio [OR] 0.632, 95% confidence interval [CI] 0.40 to 0.97; *P* = 0.044). After adjusting for confounding factors, extraocular muscle limitation and TSI were associated with the IVMP response. Less extraocular muscle limitation (OR 0.463, 95% CI 0.25 to 0.85, *P* = 0.014) and high TSI level were associated with a high risk of poor IVMP treatment response (OR 1.005, 95% CI 1.000 to 1.009, *P* = 0.038).

**Table 2:** Factors associated with the response to the steroid treatment investigated by univariate and multivariate binary logistic regression analysis (for an unresponsive group).

Univariate analysis	P-value	Odds ratio	95% confidence interval				
			Lower		Upper		
**Age**	0.909	1.002	0.965		1.041	
**Sex**	0.637	1.289	0.448		3.709	
**BMI**	0.459	1.076	0.887		1.305	
**Thyroid eye disease type (fat dominant: muscle dominant)**	0.792	1.167	0.371		3.678	
**Graves’ disease duration**	0.595	0.997	0.987		1.007	
**Thyroid eye disease duration**	0.254	0.970	0.920		1.022	
Smoking status							
Never smoker	0.318	(ref)				
Ex-smoker	0.999	0.999	0			
Current smoker	0.130	0.352	0.091		1.360	
CAS							
3	0.637	(ref)				
4	0.173	2.545	0.664		9.751	
5	0.176	2.909	0.620		13.651	
6	0.314	2.667	0.396		17.977	
7	1.000	0	0			
Gorman							
No diplopia	0.260	(ref)				
Intermittent diplopia	0.329	0.439	0.084		2.288	
Inconstant diplopia	0.467	0.537	0.101		2.862	
diplopia	0.063	0.220	0.045		1.084	
**Exophthalmos measurement (OD)**	0.181	1.132	0.944		1.357	
**Exophthalmos measurement (OS)**	0.088	1.197	0.973		1.471	
**Difference in proptosis**	0.236	0.724	0.424		1.235	
**Extraocular muscle limitation**	^*^0.044	0.632	0.405		0.987	
**Visual field index (OD)**	0.312	0.981	0.945		1.018	
**Visual field index (OS)**	0.577	0.991	0.959		1.023	
**Free T4**	0.647	0.799	0.306		2.086	
**TSH**	0.234	1.029	0.982		1.078	
**T3**	0.848	0.999	0.993		1.006	
**TSH receptor antibody**	0.280	1.020	0.984		1.059	
**TSI**	0.112	1.003	0.999		1.006	
**Total cholesterol**	0.937	0.999	0.985		1.014	
**LDL cholesterol**	0.497	0.994	0.978		1.011	
**HDL cholesterol**	0.334	1.012	0.988		1.037	
**Triglyceride**	0.166	0.995	0.987		1.002	
Multivariate analysis							
**Extraocular muscle limitation**	^*^0.014	0.463	0.251		0.855		
**TSI**	^*^0.038	1.005	1.000		1.009		
CAS							
3	0.377	(ref)					
4	0.208	2.620	0.584		11.748		
5	0.064	5.913	0.899		38.898		
6	0.148	5.894	0.534		65.031		
7	1.000						
Smoking status							
Never smoker	0.541	(ref)					
Ex-smoker	0.998	0.000	0.000				
Current smoker	0.268	0.415	0.088		1.965		

BMI, body mass index; CAS, clinical activity score; TSH, thyroid-stimulating hormone; TSI, thyroid-stimulating immunoglobulin; LDL, low-density lipoprotein; HDL, high-density lipoprotein; OD, oculus dexter; OS, oculus sinister.

^*^Statistically significant values with p <0.05.

Model chi-squared test p=0.001, Nagelkerke R2 = 0.387, Hosmer and Lemeshow test p=0.707.

### 3.3 FS

Based on the results shown in **Table 1**, the least associated variables of age, height, type of TED, symmetry, vision, total cholesterol, and T3 were excluded from the original 32 variables in the first FS method (*P* = 0.910, *P* = 0.933, *P* = 0.791, *P* = 0.729, *P* = 0.959, *P* = 0.938, and *P* = 0.806, respectively). Second, we selected the final 17 top-ranked features (TSI, low-density lipoprotein [LDL] cholesterol, body mass index [BMI], TSH, Gorman score, Tg, TSHR ab, Visual field (VF) index, weight, high-density lipoprotein cholesterol, difference in exophthalmos, free T4, exophthalmos, duration of Graves’ disease, and CAS) based on the random forest and XGBoost feature importance methods.

### 3.4 Results of XGBoost classifier

We analyzed the features to predict responsiveness to IVMP treatment using both the random forest classifier and XGBoost. Before we applied SMOTE, the cross-validation accuracies of both models were 0.84 and 080. Using SMOTE, 142 datasets were created, and the final performance of XGBoost is presented in **Table 3**. When evaluated using the confusion matrix, the relatively high false-positive of 14 cases in the first model and 82.9% positive predictive value (PPV) before applying SMOTE were noticeably improved after using SMOTE. The false-positive of the model after applying SMOTE were 8 and PPV was 88.8%.

**Table 3:** Performance of models.

	CV mean accuracy		Standard deviation
**Random Forest**	0.805		0.282
**Linear Support vector machine**	0.709		0.140
**KNN**	0.776		0.084
**Decision Tree**	0.673		0.106
**Naïve Bayes**	0.688		0.155
**Logistic regression**	0.698		0.163
**XGboost**	0.808		0.101
**XGboost (SMOTE)**	0.861		0.093
	XGBoost	XGboost (SMOTE)	P-value
**CV mean accuracy**	0.808	0.861	0.019

XGBoost model showed higher accuracy than other classic machine learning algorithm and traditional statistical algorithm. After using SMOTE, the mean CV accuray of XGBoost was highly improved. Ten-fold cross-validation was applied for these algorithms and mean CV accuracies were expressed due to its shortage of dataset.

CV cross-validation; KNN k-nearest neighbor; SMOTE synthetic minority oversampling technique

### 3.5 Important features predicting responsiveness after IVMP treatment investigated by SHAP

**Figure 3:**
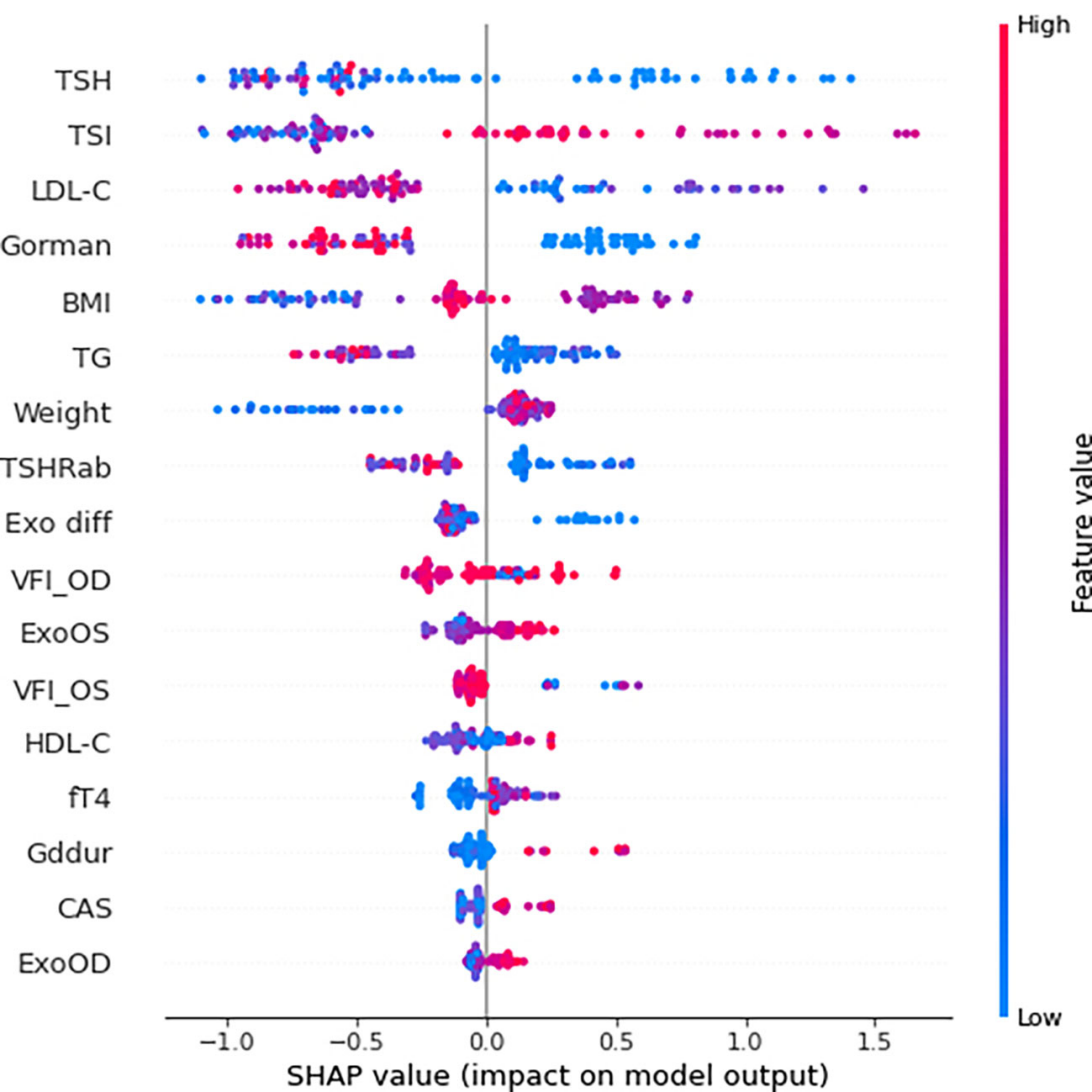
SHAP summary plot of feature importance to predict steroid responsiveness. SHAP, SHapley Additive exPlanations; TSH thyroid-stimulating hormone; TSI thyroid-stimulating immunoglobulin; LDL-C low-density lipoprotein cholesterol; BMI body mass index; TG triglyceride; TSHRab thyroid-stimulating hormone receptor antibody; Exo diff differences of exophthalmos; VFI_OD visual field index_oculus dexter; ExoOS exophthalmos in oculus sinister; VFI_OS visual field index_oculus sinister; HDL-C high-density lipoprotein cholesterol; Gddur disease duration of graves disease; CAS clinical activity score; ExoOD exophthalmos in oculus dexter.

**Figure 4:**
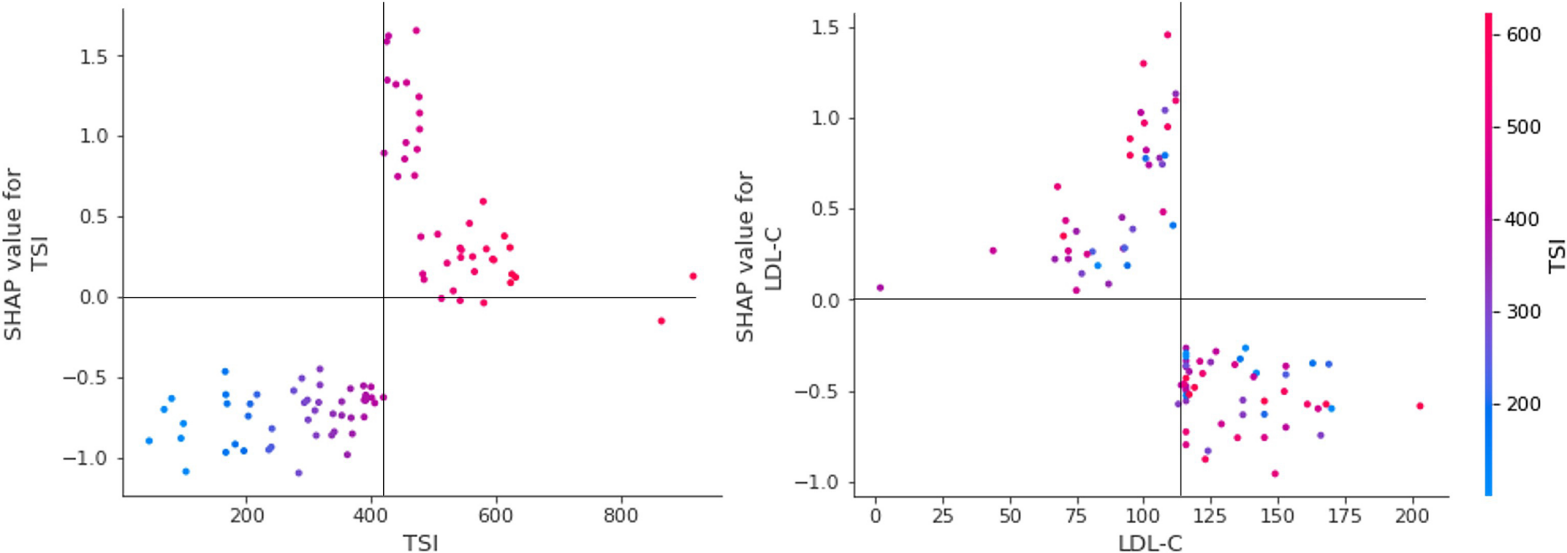
SHAP dependence plot for essential features. SHAP dependency analysis of TSI on the left and SHAP dependency analysis of LDL-cholesterol is described on the right. SHAP, SHapley Additive explanation; TSI thyroid-stimulating immunoglobulin; LDL-C low-density lipoprotein cholesterol.

The SHAP summary results are shown in **Figure 3**, which rank features according to their importance in predicting responsiveness to IVMP treatment. TSH, TSI, and LDL cholesterol levels had the greatest effects on the model. Among them, TSI and LDL cholesterol showed a specific pattern in which a higher TSI level was associated with a significant impact on the model and a lower LDL cholesterol level was associated with a significant impact on the model. **Figure 4** shows the effect of TSI and LDL cholesterol on responsiveness. TSI levels lower than 400 decreased the predicted probability, while a high level of TSI increased the predicted treatment responsiveness probability. An LDL cholesterol level of 80–100 increases the predicted probability, and an LDL cholesterol level over 110 decreases the predicted probability. TSH showed no definite pattern on SHAP dependence analysis.

## 4 Discussion

In this study, we discovered that severe extraocular movement limitation indicated a better response to IVMP treatment. Concurrently, higher TSI levels have been linked to a poor response to IVMP treatment in patients with active moderate-to-severe TED. Ahn and Lee (16) reported that Extra ocular muscle(EOM) enlargement on CT is a predictor of a favorable steroid treatment response, and Xu et al. found that the thickness of the EOM on magnetic resonance imaging was considerably higher in the responsive group than in the unresponsive group. Moreover, Naik et al. (17) classified soft tissue involvement in TED as either fat growth or predominant muscle enlargement, with the latter being more susceptible to steroid treatment. Inflammation and edematous changes in the EOM cause EOM enlargement and limit motility, which is consistent with the results of several other studies. In the multivariate analysis excluding interference from other factors, we found an OR of 0.463 (p = 0.014) for patients with severe extraocular movement limitation in response to treatment. However, this finding does not indicate better extraocular movement after treatment, and the overall inflammatory symptoms resolved. Additional research is required to determine extraocular movements based on treatment.

Although there is limited research regarding TSI, in 1995, Mori et al. reported that TSAb could be a valuable marker for predicting the efficacy of methylprednisolone pulse therapy in TED patients. In nine TED patients treated with intravenous methylprednisolone in this study, they found that high levels of TSAb are expected to be a factor for good response to therapy. Conversely, our study showed that a higher TSI level was significantly associated with poor responsiveness to treatment (OR, 1.005; p = 0.038) in the multivariate analysis. Furthermore, the TSI level analyzed with XGBoost was one of the most important features for predicting treatment responsiveness.

In terms of model interpretation, we applied SHAP to obtain a compelling and insightful measure of the importance of a feature in a model (18). SHAP is a method for estimating the contribution of each factor or characteristic, and it breaks down the prediction to show the impact of each feature (19). According to our SHAP results, we found specific patterns of TSI and LDL cholesterol levels. High TSI values over 400 and lower LDL cholesterol values under approximately 120 mg/dL enhanced the model prediction and significantly impacted the model. Both of these were the top two important features analyzed with permutation feature importance in the FS process.

The TSI has been reported as an excellent marker in patients with TED because of its sensitivity and specificity (20). TED was observed in TSI-positive but TRab-negative participants in one controlled trial, and TSI had a striking positive connection with the clinical activity and severity of TED. They also found that TSI causes TED pathogenesis even after anti-thyroid medication, and that TSI positivity was low in Graves’ disease(GD) patients without TED. Moreover, several studies have emphasized the need for TSI monitoring throughout the disease follow-up and treatment (21, 22). From this point of view and from our research, TSI is useful for diagnosis and follow-up, and for predicting steroid therapy response prior to treatment. According to our study’s SHAP analysis of TSI, when the TSI level exceeds approximately 400%, the impact on model output increases, and 400% serves as a critical pivot point for predicting responsiveness to IVMP treatment. When combined with statistical analyses, it is reasonable to assume that a TSI of 400% or above does not respond well to treatment. However, it is noteworthy that the AI model does not predict the outcome on the basis of this single attribute.

Several studies have been conducted to determine the relationship between lipid-lowering agents, LDL-cholesterol, and TED. According to Stein et al. (23), patients treated with statins had a 40% decreased risk of developing TED because of the anti-inflammatory action of statins. Sabini et al. (24) found a strong association between the presence of TED and both total and LDL cholesterol in patients with newly diagnosed GD. Additionally, there is a considerable direct link between TED and LDL cholesterol levels. LDL cholesterol levels were shown to be considerably higher in individuals with TED, and cutoff values for LDL cholesterol were established at 118.4 mg/dL, with levels above these values significantly related to an elevated risk of TED. Surprisingly, a value of 115 mg/dL for LDL cholesterol in the artificial intelligence analysis in our study revealed a critical insight. Although LDL did not demonstrate any statistical significance in statistical analyses, including multiple regression, it had the greatest influence on the construction of the AI model; when the value was less than the reference point near 115–120 mg/dL, it had a higher impact on the model’s prediction. Additional inflammation caused by a high LDL cholesterol may be taken to suggest that it adversely affects the prediction of steroid response, but once again, it is unreasonable to anticipate a patient’s treatment response in the clinic based on this factor (25, 26).

Finally, a final XGBoost model with high performace was constructed using 17 factors, and a more trustworthy and useful model was constructed using the SMOTE technique to overcome the high false-positive rate.

## 5 Conclusion

Consequently, statistically, EOM limitation and TSI level had a significant effect on the prediction of treatment response, and among the factors proven using machine learning, TSH, TSI, and LDL-C were the top three features; however, in the case of TSH, there were difficulties in the interpretation.

Although the number of patients was limited and the study was retrospective, this is the first study to validate the steroid response factor *via* XGBoost. We believe that this study verifies the applicability of the gradient boosting model to a variety of ophthalmic diseases by demonstrating that noteworthy results were obtained.

## Data availability statement

The data analyzed in this study is subject to the following licenses/restrictions: Due to its ethical concerns, supporting data cannot be made openly available. further inquiries can be directed to the corresponding author. Requests to access these datasets should be directed to Jungyul Park, ophjyp@naver.com.

## Ethics statement

The studies involving human participants were reviewed and approved by The Institutional Review Board (IRB) of Pusan National University Hospital (IRB No. 2112-006-109), South Korea. Owing to the retrospective nature of the study, the IRB waived the need for patient consent. Written informed consent for participation was not required for this study in accordance with the national legislation and the institutional requirements.

## Author contributions

Conception and design of the study (JP, JK, HC); conduction of the study (JP, JK, HC); collection and management of data (JP, JK); data analysis (JP, JK); data interpretation (JP, D-MR, JK, HC); and preparation, review, and approval of the manuscript (JP, JK, D-MR, HC).
